# Paraneoplastic pemphigus regression after thymoma resection

**DOI:** 10.1186/1477-7819-6-83

**Published:** 2008-08-12

**Authors:** Nikolaos Barbetakis, Georgios Samanidis, Dimitrios Paliouras, Ioannis Boukovinas, Christos Asteriou, Eleni Stergiou, Kostas Laschos, Christodoulos Tsilikas

**Affiliations:** 1 Thoracic Surgery Department, Theagenio Cancer Hospital, Thessaloniki, Greece; 2 Second Department of Clinical Oncology, Theagenio Cancer Hospital, Thessaloniki, Greece; 3 First Department of Clinical Oncology, Theagenio Cancer Hospital, Thessaloniki, Greece

## Abstract

**Background:**

Among human neoplasms thymomas are associated with highest frequency with paraneoplastic autoimmune diseases.

**Case presentation:**

A case of a 42-year-old woman with paraneoplastic pemphigus as the first manifestation of thymoma is reported. Transsternal complete thymoma resection achieved pemphigus regression. The clinical correlations between pemphigus and thymoma are presented.

**Conclusion:**

Our case report provides further evidence for the important role of autoantibodies in the pathogenesis of paraneoplastic skin diseases in thymoma patients. It also documents the improvement of the associated pemphigus after radical treatment of the thymoma.

## Introduction

Paraneoplastic pemphigus is rarely associated with thymic neoplasms either alone or concomitantly with other autoimmune disorders. A case of a 42-year-old woman with paraneoplastic pemphigus as the first manifestation of thymoma is reported. Transsternal complete thymoma resection achieved pemphigus regression. The pathogenetic and clinical relationships between pemphigus and thymoma encountered in this case are presented with the purpose of strengthening the hypothesis that pemphigus in the presence of a thymoma could be a thymoma-associated autoimmune disease.

## Case presentation

**Figure 1:**
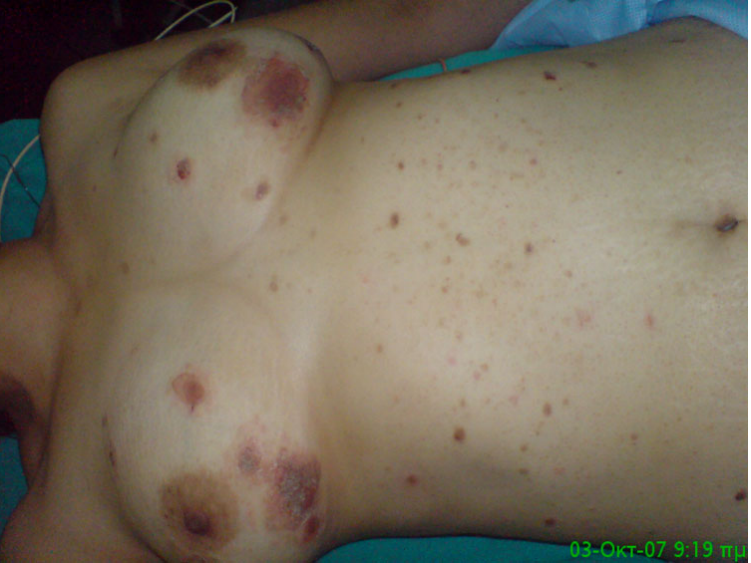
**Numerous crusted, denuted and flaccid vesiculobullous lesions over the chest and abdomen consistent with pemphigus**.

**Figure 2:**
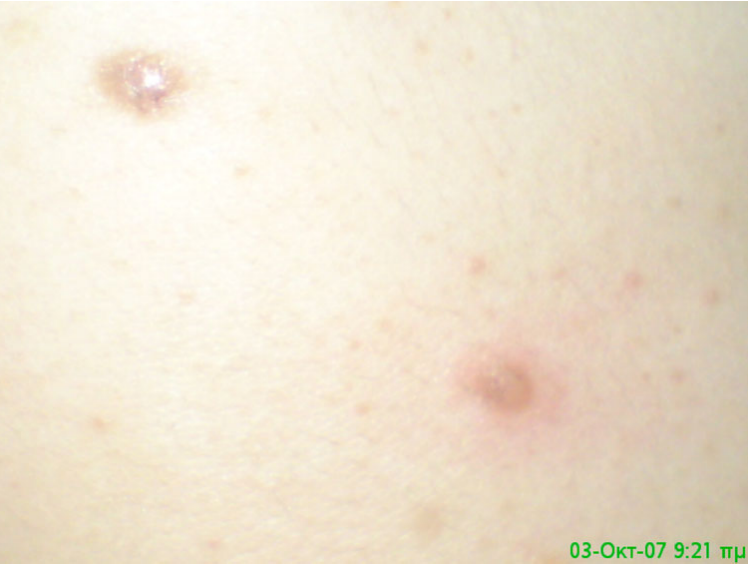
**Close view of a pemphigus lesion**.

A 42-year-old otherwise healthy woman was admitted to our hospital with numerous crusted, denuted and flaccid vesiculobullous lesions over her chest, abdomen, legs, toes and forehead (Figures 1, 2). Vesicles were also observed on the buccal mucosa and palate. The clinical diagnosis of pemphigus vulgaris was confirmed by biopsy. Histological findings included interface dermatitis, apoptotic keratinocytes and focal areas of suprabasal acantholysis. Therapeutic management consisted of successive cycles of 50 mg/day of prednisone. Medical treatment provided only transient relief of symptoms and temporary reduction in clinical manifestations and on interruption of systemic steroid the lesions reccured.

**Figure 3:**
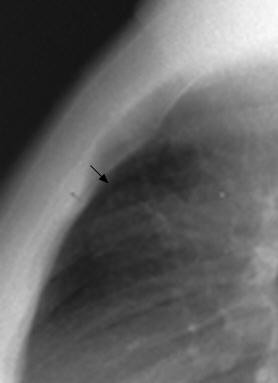
**A profile chest x-ray showing a retrosternal tumor**.

**Figure 4:**
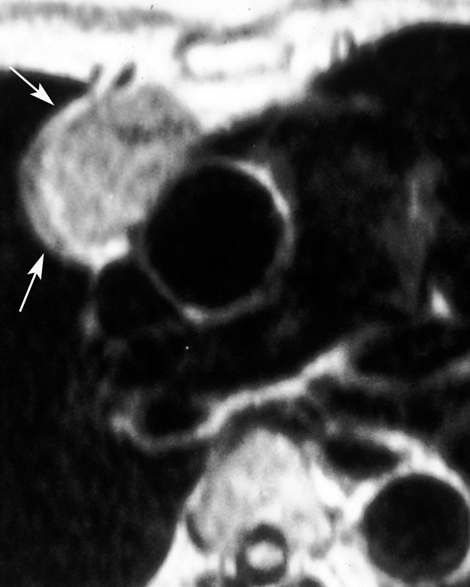
**MRI of the chest revealed an anterior mediastinal mass consistent with a thymic tumor**.

Three months later while facing a persistent pemphigus, a chest x-ray was done and a retrosternal tumor was noted (Figure 3). CT scans and MRI of the chest revealed a homogeneous anterior mediastinal mass consistent with a thymic tumor but with no lymph node involvement (Figure 4). In order to perform preoperative staging of the tumor, the patient underwent CT scans of brain, abdomen and a bone scan. All were normal. An autoimmune laboratory profile proved that desmoglein-1 (anti-DSG-1) antibodies were normal while the desmoglein-3 (anti-DSG-3) titer reached twice the normal.

**Figure 5:**
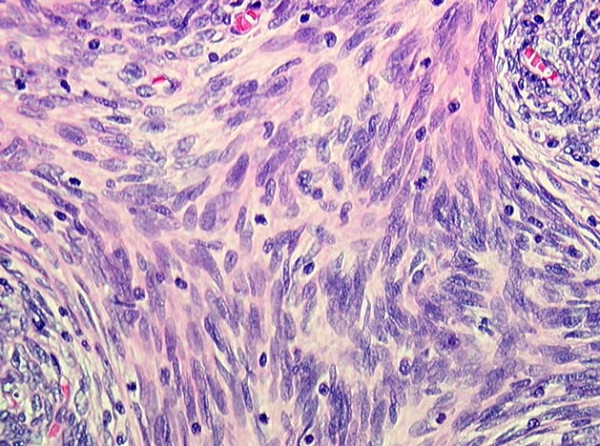
**Thymic tumor consisting of spindle cells**.

**Figure 6:**
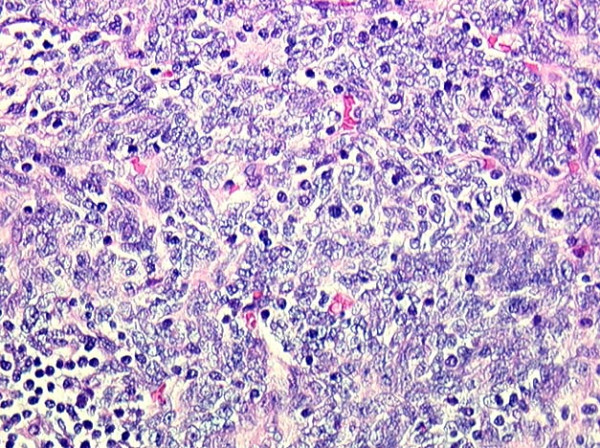
Varying numbers of lymphocytes with rounder epithelial cells populating spindle cells consisting with a non invasive thymoma.

At operation through a median sternotomy, a solid intrathymic capsulated mass was found and a thymectomy was performed. Definitive pathologic examination found a tumor measuring 6 × 5 × 5 cm occupying the inferior part of the thymus gland consisting of spindle cells populated by varying numbers of lymphocytes with rounder epithelial cells (Figures 5, 6). No capsular involvement was noted (Type A non invasive thymoma according to World Health Organization classification).

The postoperative period was uneventful. The patient was discharged home after 10 days Postoperatively anti-DSG-1 and anti-DSG-3 showed a transient increase. At the outpatient visit four weeks later both desmoglein antibodies showed a significant decrease associated with almost complete resolution of the cutaneous lesions. Twelve months later she is still disease free and with no need of steroids.

## Discussion

**Figure 7:**
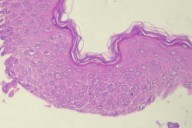
Microscopic appearance of pemphigus with epithelial cells falling away from each other and rounding out in the fluid of the blister.

The relation between thymus disease and autoimmune disorders is well known and established [1]. In 1987 a retrospective study among 172 patients with thymoma found fungal mucocutaneous disease as the most common thymoma-associated cutaneous disorder [2]. Two patients in that study were noted with pemphigus and lichen planus respectively. Since then paraneoplastic pemphigus has been recognized as a well defined autoimmune syndrome characterized by autoantibody formation directed against epithelial antigens [3]. In our case pemphigus was diagnosed first by biopsy (Figure 7) and this has led us to further diagnostic investigation.

Serial analysis of various case reports correlate pemphigus with thymoma and/or myasthenia gravis although in our case there were no symptoms or signs of myasthenia gravis [4,5]. The clinical course and titrations of antiepithelial, antimuscle, and antithymus antibodies suggested a reverse relationship between the severity of myasthenia gravis and titers of antimuscle and antithymus antibodies, and a parallel relationship between pemphigus vulgaris and antiepithelial antibody.

The association of paraneoplastic pemphigus with malignancy is strong. Only a handful of patients have had no associated diagnosis. Some patients have had benign neoplasms, including as in our case thymoma or Castleman's disease. Only a single patient without a tumor has met the diagnostic criteria, yet this patient had a rapid demise and may have died with an undiagnosed malignancy [6]. Patients have developed paraneoplastic pemphigus while in remission of their malignancy, leading some authors to prefer the term neoplasia-induced pemphigus.

Treatment of the underlying malignancy does not necessarily halt progression of the paraneoplastic pemphigus, although some have observed that clinical manifestations improve as autoantibody titers decrease following resection of the tumor. This was also proved in our case, where anti-DSG-3 showed a significant decrease associated with almost complete resolution of the cutaneous lesions. Circulating and tissue-bound antibodies in patients with this disease are directed against a group of molecules with sequence homology and belonging to the plakin family. These molecules are found in the intracellular attachment plaques of desmosomes and hemidesmosomes, and they play a key role in intermediate filament attachment. However, the number of reported target antigens has increased over time and varies between patients. This variability likely accounts for the clinical heterogeneity of this disease. By immunoprecipitation, target antigens (in decreasing order of incidence) include desmoglein 3, desmoglein 1, envoplakin (210 kd), periplakin (190 kd), desmoplakin I (250 kd), desmoplakin II (210 kd) and bullous pemphigoid antigen I (230 kd). Plectin (> 400 kd) and an unidentified 170-kd protein have also been found [7].

In our case the levels of pemphigus-associated antibodies had increased postoperatively after thymectomy. In parallel, decreased titer of those antibodies preceded the resolution of cutaneous lesions. A similar beneficial effect of tumor ablation has recently also been reported in the case of Castleman's tumor, a rare lymphoproliferative disease that is sometimes associated with paraneoplastic pemphigus [8].

## Conclusion

our case report provides further evidence for the important role of autoantibodies in the pathogenesis of paraneoplastic skin diseases in thymoma patients. It also documents the improvement of the associated pemphigus after radical treatment of the thymoma. Further studies are necessary to analyze possible pathogenetic mechanisms.

## Conflict of interests

The authors declare that they have no competing interests.

## Authors' contributions

NB, GS, DP, IB, CA, ES and KL took part in the care of the patient and contributed equally in carrying out the medical literature search and preparation of the manuscript. CT participated in the care of the patient and had the supervision of this report. All authors approved the final manuscript.
